# The Microbiome–Gut–Brain Axis, a Potential Therapeutic Target for Substance-Related Disorders

**DOI:** 10.3389/fmicb.2021.738401

**Published:** 2021-10-06

**Authors:** Xuan Fu, Ti Chen, Jingda Cai, Bo Liu, Yaohui Zeng, Xiaojie Zhang

**Affiliations:** ^1^Department of Psychiatry, The Second Xiangya Hospital, Central South University, Changsha, China; ^2^National Clinical Research Center on Mental Disorders, Changsha, China; ^3^National Technology Institute on Mental Disorders, Changsha, China; ^4^Hunan Medical Center for Mental Health, Changsha, China; ^5^Hunan Key Laboratory of Psychiatry and Mental Health, Changsha, China; ^6^Mental Health Institute of Central South University, Changsha, China; ^7^Department of Laboratory Medicine, The Third Xiangya Hospital, Central South University, Changsha, China

**Keywords:** microbiome-gut-brain axis (MGBA), microbiota manipulation, substance-related disorders, gut microbiota, fecal microbiome transplantation

## Abstract

Substance addiction is a complex worldwide public health problem. It endangers both personal life and social stability, causing great loss on economy. Substance-related disorder is considered to be a complicated chronic brain disorder. It resulted from interactions among pharmacological properties of addictive substances, individual susceptibility, and social–environmental factors. Unfortunately, there is still no ideal treatment for this disorder. Recent lines of evidence suggest that gut microbiome may play an important role in the pathogenesis of neuropsychiatric disorders, including substance-related disorders. This review summarizes the research on the relationship between gut microbiome and substance-related disorders, including different types of substance, different individual susceptibility, and the occurrence and development of substance-induced mental disorders. We also discuss the potentiation of gut microbiome in the treatment of substance-related disorders, especially in the treatment of substance-induced mental disorders and manipulation on individuals’ responsiveness to addictive substances.

## Introduction

Substance addiction is a complex worldwide public health problem. According to the *2020 World Drug Report*, under the COVID-19 pandemic, a global health menace caused by substance abuse becomes more and more complicated and brings tremendous threats to social security and economy (WHO, 2020), such as the global opioid crisis, the rapidly spreading synthetic substance market, the legalization of non-medical marijuana, and the supply chains on the dark web. In addition to illegal drugs, addiction of tobacco and alcohol also had huge impact on human health. Substance-related disorder in DSM-5 refers to a cluster of cognitive, behavioral, and physiology symptoms related to the continuous use of substances, encompassing substance use disorder, intoxication, withdrawal, other substance-induced disorders, and unspecified substance-related disorders (Saunders, 2017). The reward circuit, ventral tegmental area–nucleus accumbens (VTA–NAc) circuit (Kim et al., 2016), is considered to be the structural basis of substance-related disorders (Koob and Volkow, 2016). Long-term drug abuse leads to abnormal activation of dopaminergic neurons in reward circuits (Koob and Le Moal, 2001). However, the mechanism of substance-related disorders is unclear, and more importantly, treatments with comparable efficacy and tolerable side effects for these disorders are still under development.

In recent decades, the impact of gut microbiome on the nervous system has aroused extensive interest. Studies show that there is a bidirectional communication system between gut microbiome and brain, which is known as the microbiome–gut–brain axis (MGBA) (Dinan and Cryan, 2017). Increasing evidence revealed the role of intestinal microbial community in neuropsychiatric diseases (Burokas et al., 2015), including reward process and substance use disorders (Garcia-Cabrerizo et al., 2021). It has been found that the composition and diversity of gut microbiome in substance-addicted individuals changed remarkably (Meckel and Kiraly, 2019). A study showed the specific impacts of sex in the correlation of addictive related behaviors and gut microbiome according to comprehensive behavioral characterization and gut microbiome analysis in rats (Peterson et al., 2020). It was also becoming clear the impact of the microbiome–gut–brain axis on hypothalamic–pituitary–adrenal (HPA) axis stress response and depression, which were all risk factors for substance use disorders. The underlying mechanisms were explored not only in the overall modulation of immune responses and intestinal barrier integrity, but also in the neurotransmitter synthesis and metabolism, such as serotonin and dopamine (Leclercq et al., 2014a; Skosnik and Cortes-Briones, 2016). Recently, “psychobiotic” treatment strategies had great promise as novel treatment for drug addiction (Skosnik and Cortes-Briones, 2016). Here, we reviewed the preclinical and clinical studies of correlation between intestinal microbial community and different addictive substances (alcohol, opioid, cocaine, amphetamine, etc.) and then focused on the role of gut microbiome in individual susceptibility to addictive substances and substance-related mental disorders. We also discussed the therapeutic potential of gut microbiome in the treatment of substance-related disorders, especially in the treatment of substance-induced mental disorders and manipulation on individuals’ responsiveness to addictive substances. However, further systematic investigations were required to expand towards casual and mechanistic relationships between gut microbiome and drug addiction (Temko et al., 2017). More basic researches and clinical trials were needed to examine the efficacy of probiotic or combination treatments.

## The Role of the Microbiome–Gut–Brain Axis in Alcohol Use Disorders (AUD)

Alcohol directly affects intestinal micro-ecology. Both animal and clinical studies had shown that alcohol can cause changes in the diversity and composition of the gut microbiome (Bajaj, 2019; Wang S. C. et al., 2020), which induced intestinal mucosal damage and increases intestinal permeability in some patients (Leclercq et al., 2014b; Peterson et al., 2017). As intestinal permeability increases, intestinal bacteria and metabolites including neurotransmitters produced by gut microbiome may enter the peripheral blood circulation and affect the host’s health (Leclercq et al., 2014b; Mayer et al., 2014; Szabo, 2015).

Although the impact of alcohol on gut microbiome was clear, as we summarized in Table 1, the effects of alcohol on gut microbiome and its metabolites were diverse in different studies. In preclinical studies, firstly, different species, mice, rats, and rhesus macaques, showed different gut microbiome responses to alcohol (Yan et al., 2011; Bull-Otterson et al., 2013; Barr et al., 2018; Fan et al., 2018; Wang G. H. et al., 2018). Even though the same mouse models were employed, alcohol administration was different, namely, 5% ethanol Lieber-DeCarli pair-fed diet daily feeding (Lowe et al., 2017), chronic intermittent vaporized ethanol for 4 weeks (Peterson et al., 2017), and fermented rice liquor consumption (Lee et al., 2020), which showed diverse alteration in gut microbiome. One study of rhesus macaques showed that the voluntary alcohol self-administration for 12 months did not alter the microbial community diversity (Barr et al., 2018), while another study showed that microbiome change induced by 3-month alcohol consumption can be reversed by 5-day withdrawal (Zhang X. et al., 2019). In one clinical study that compared with 24 healthy controls, the number of *Bifidobacteria*, *Lactobacilli*, and Enterococci were significantly reduced in 66 alcoholic patients (Kirpich et al., 2008). Patients with alcohol use disorder presented a unique pattern of gut microbiome: *Akkermansia* decreased and *Bacteroides* increased, with an accuracy of 93.4% (Addolorato et al., 2020). In alcoholics with dysbiosis, the median abundance of Proteobacteria was lower, which correlated with high levels of serum endotoxin in some subjects, and they had a higher frequency of mild diabetes (Mutlu et al., 2012). Patients diagnosed with alcohol dependence had higher intestinal permeability and their gut microbiome profile was altered, with more microbiome from Lachnospiraceae and Incertae Sedis XIV and less from Ruminococcaceae and Incertae Sedis XIII at the family level. The family Erysipelotrichaceae and the genus *Holdemania* decreased significantly after 3 weeks of detoxification, compared to the beginning of withdrawal (Leclercq et al., 2014b). In summary, although preclinical and clinical studies were complicated by many factors, including different species, alcohol concentration, types of ferment, stages of alcohol use disorders (AUD), mode and duration of alcohol administration, and the duration of withdrawal, there was still evidence to support the safety and benefit of microbial manipulation in short-term alcohol craving and consumption. However, further trials with larger number of patients are needed.

**TABLE 1:** Effects of alcohol on gut microbiome and other systems.

Species	Alcohol administration	Sample size	Effect on microbiome and microbial metabolites	Effect on other systems	Citations
Human	Alcoholic About 1/3 alcoholics dysbiotic	Alcoholics with liver disease *n* = 19 Alcoholics without liver disease *n* = 29 Control group *n* = 18	Mucosa-associated microbiome ↓*Bacteroidetes* ↑*Proteobacteria*	↑Serum endotoxin ↑Frequency of mild diabetes	Mutlu et al., 2012
	Alcohol use disorder (AUD)	AUD patients *n* = 36 (14 with cirrhosis) Control *n* = 36	↓*Akkermansia* ↑*Bacteroides*	↑LPS, TNFα, IL-6 ↑GABA metabolic pathways and energy metabolism	Addolorato et al., 2020
	Acute alcohol binge	Healthy individuals (11 males and 14 females)	↑In serum bacterial translocation from the gut	↑Serum endotoxin ↑TNFα, IL-6, chemokine, MCP-1	Bala et al., 2014
	AD patients	AD patients with cirrhosis *n* = 27 AD patients without cirrhosis *n* = 72	↓The levels of butyrate-producing species from the Clostridiales order ↑Functional microbiome pathways related to alcohol metabolism and inflammation	↑Biotransformation of bile acids ↑Gut permeability ↑Acetaldehyde	Dubinkina et al., 2017
	AD patients	AD patients *n* = 40 Control group *n* = 16	↑Intestinal permeability and blood Lipopolysaccharides at onset of withdrawal but returned to normal in the end	Low-grade inflammation ↑TNF-α, IL-6 and IL-10	Leclercq et al., 2012
		Actively drinking noncirrhotic alcohol-dependent patients *n* = 63 Control group *n* = 18	↑Lipopolysaccharide- and peptidoglycan-associated receptors	↑Messenger RNA and plasma levels of interleukin (IL)-8, IL-1β, and IL-18	Leclercq et al., 2014a
	Alcoholic cirrhosis with active drinking or abstinent	Healthy control individuals *n* = 34 Actively drinking patients with cirrhosis *n* = 37 Abstinent patients with cirrhosis *n* = 68	Dysbiosis was prevalent in all tissues studied in actively drinking patients with cirrhosis	↑Secondary and glycine-conjugated Bas ↑Endotoxemia, systemic and ileal inflammatory expression, and lower amino acid and bioenergetic-associated metabolites	Bajaj et al., 2017
Rat	Chronic alcohol consumption/Alcohol-dependent	Alcohol-dependent rats *n* = 20 Control group *n* = 20	↓*Lactobacillus* and *gauvreauii* ↑*Bacteroidetes* No significant effect on diversity and richness of gut microbiome	↑Amino acid metabolism, polyketide sugar unit biosynthesis and peroxisome	Yang et al., 2017
	Daily trained for 30 min to self-administer 0.1 ml of alcohol 10% weight/volume for 80 consecutive sessions	Based on addiction criteria scores Vulnerable group *n* = 19 Resilient group *n* = 40	↑Clostridiales, Ruminococcaceae, Lachnospiraceae ↓*Desulfovibrio*, *Gemella*, Coriobacteriaceae, *Hydrogenoanaerobacterium*	D2R mRNA expression positively correlated with *Firmicutes*, negatively correlated with *Veillonella*, *Gemella*, and Ruminococcaceae	Jadhav et al., 2018
	10 days of 5% alcohol daily feeding	Jejunum Control *n* = 6 Alcohol dependent *n* = 6 Alcohol withdrawal *n* = 6 Colon Control *n* = 6 Alcohol dependent *n* = 6 Alcohol withdrawal *n* = 6	Alpha- and beta-diversity of gut microbiome are not altered in the jejunum and colon. In the colon, alcohol dependence group compared with the withdrawal and control groups: ↑*Phyla Bacteroidetes* ↑*Bacteroidales* S24-7, Ruminococcaceae, *Parabacteroides*, *Butyricimonas* ↓*Lactobacillus* and *gauvreauii*	NA	Fan et al., 2018
Mouse	Chronic alcohol consumption/alcohol-dependent	Active drinking group *n* = 10 Forced drinking group *n* = 10 Control group *n* = 10	↓Lachnospiraceae, *Alistipes*, and *Odoribacter* ↑*Firmicutes* and diversity of gut microbiome	↑Serotonin and bile acids ↑Anxiety and depression	Wang F. et al., 2018
		Alcohol-fed group *n* = 8 Pair-fed group 10	↑*Actinobacteria* ↓*Tenericutes* ↓*Verrucomicrobia*/*Akkermansia*	↑Serum endotoxin ↑Alcohol-induced inflammatory mediators in the liver ↑Neutrophil infiltration to the liver	Lowe et al., 2017
		Alcohol-dependent mice *n* = 8 control group *n* = 8	↓*Bacteroidetes*, *Firmicutes* ↑*Actinobacteria*, *Proteobacteria*, and blood LPS	↑Plasma endotoxin, fecal pH, hepatic inflammation, and injury	Bull-Otterson et al., 2013
		Alcohol-fed mice *n* = 5 control group *n* = 5	↑*Bacteroidetes*, *Verrucomicrobia*	↓Expression of bactericidal c-type lectins Reg3b and Reg3g	Yan et al., 2011
	Chronic intermittent vaporized alcohol exposure	Experimental group *n* = 10 Control group *n* = 9	↓*Clostridium IV, Clostridium XlVb*, *Dorea*, and *Coprococcus* ↑*Alistipes*	NA	Peterson et al., 2017
	Low-dose and short-term (7 days) alcohol or fermented rice liquor (FRLs) consumption	1 vehicle group with alcohol, 3 FRLs groups with same dose of alcohol, 1 control group (*n* = 9–12)	↓*Bacteroidetes* in alcohol group ↑*Firmicutes* in alcohol group but recovered in FRLs groups	↑Fecal production of SCFA FRLs consumption reduces alcohol-induced inflammatory responses	Lee et al., 2020
Rhesus macaques	Freely drinking alcohol Adolescent 3 months/Adult 5 years	Adolescent alcohol group *n* = 6 Adolescent control group *n* = 6 Adult alcohol group *n* = 4 Adult control group *n* = 5	↓Diversity ↓Verrucomicrobia and Proteobacter ↑*Firmicutes*	Changes in glycolysis and fatty acids ↓Ursolic acid ↓Isobar dihydrocaffeate	Zhang X. et al., 2019
	Voluntary ethanol self-administration 12 months	Heavy drinkers *n* = 4 Non-heavy drinkers *n* = 4 Control group *n* = 4	No difference in microbial community diversity at the group level	Changes in gene expression of protein trafficking, metabolism, inflammation, and colorectal cancer development	Barr et al., 2018

## The Role of the Microbiome–Gut–Brain Axis in Alcohol-Induced Liver Damage (ALD)

Alcohol mainly metabolized in liver. So far, a large number of studies have confirmed the important role of gut–liver axis in ALD (Bajaj, 2019). Fecal microbiome transplantation (FMT) from alcohol-resistant mice into alcohol-sensitive mice or pectin prebiotics can reshape intestinal micro-ecology homeostasis and prevent alcohol-induced fatty liver and inflammation (Ferrere et al., 2017). Specifically, a decrease in *Lactobacillus* and *Bifidobacterium* was seen in patients with alcohol dependence, and both clinical and animal studies suggested that restoration of *Lactobacillus* and *Bifidobacterium* could reduce endotoxemia and alcohol-induced liver injury (Nanji et al., 1994; Kirpich et al., 2008). Furthermore, the loss of *Akkermansia* was also considered as an early marker of alcohol-induced gut dysbiosis (Lowe et al., 2017). Patients with alcohol use disorder presented decreased *Akkermansia* and increased *Bacteroides* (Addolorato et al., 2020). Consistently, a study found that the decrease of relative abundance of *Akkermansia* was accompanied by more severe alcoholic liver disease, and the relative abundance of *Akkermansia* was the lowest in patients with severe alcoholic hepatitis. Oral supplementation of *Akkermansia* restored the ethanol-induced *Akkermansia* depletion and alleviated the liver function damage in acute and chronic alcohol-exposed mice (Grander et al., 2018). After FMT enema from a donor rich in Lachnospiraceae and Ruminococcaceae, patients with alcohol use disorder-related cirrhosis showed decreased alcohol craving and consumption, reduced serious adverse events, and improved cognitive function (Bajaj et al., 2021). Table 2 summarized preclinical studies and clinical trials of gut microbiome-based therapies for ALD. These results suggested that intervention in the gut microbiome, such as *Akkermansia*, *Lactobacillus*, and *Bifidobacterium*, is likely to be effective targets to prevent and treat ALD. Interestingly, some bacteria such as *Clostridium* genus, had been recognized as ethanol-producing bacteria (Wiegel et al., 2006), *Clostridium -sensu stricto, -XI, -XVIII*, *Lactococcus*, *Turicibacter*, and *Akkermansia* carried the genes encoding alcohol dehydrogenase and aldehyde dehydrogenase, indicating the direct functional ability of gut microbiome in alcohol production and/or metabolism (Konkit et al., 2016; Leclercq et al., 2020). *Ruminococcus* was identified as a potential acetaldehyde accumulator that is responsible for the pathogenesis of ethanol-related colorectal cancer (Tsuruya et al., 2016). These lines of evidence also revealed promising therapeutic strategies for ALD by metabolizing alcohol and aldehyde.

**TABLE 2:** Interventions altering the gut microbiome in ALD.

Recipients	Manipulation	Change in microbiome	Change in other system	Citations
Chronic alcohol consumption (Ctrl Res, *n* = 5–8; Ctrl Sens, *n* = 9–18; Alc Res, *n* = 8–21; Alc Sens, *n* = 8–21; Ctrl FMT, *n* = 4; Alc FMT, *n* = 7; Ctrl Pectin, *n* = 6; Alc Pectin, *n* = 4)	FMT from alcohol-resistant donor mice to alcohol-sensitive receiver mice three times a week Or 6.5% pectin add in daily diet from beginning until sacrifice	Restored gut homeostasis Resulted in an IM very close to that of resistant donor mice in the sensitive recipient mice Induced major modifications of the IM	Prevented steatosis and liver inflammation	Ferrere et al., 2017
Rats were fed ethanol and liquid diet containing corn oil (Corn oil–ethanol group *n* = 6; *Lactobacilli* group *n* = 6)	*Lactobacillus* daily feeding for a month	NA	↓Endotoxemia and alcohol-induced liver injury	Nanji et al., 1994
Hospitalized Russian male patients with alcoholic psychosis (Standard therapy group *n* = 34; Probiotic therapy group *n* = 32)	Probiotic (*Bifidobacteria*, *Lactobacilli*) oral supplementation for 5 days	Restoration of the bowel flora ↑*Bifidobacteria* ↑*Lactobacilli*	↓ALT, AST, GGT, lactate dehydrogenase, and total bilirubin	Kirpich et al., 2008
Female WT mice were fed a Lieber-De-Carli diet containing 1–5 vol% concentration accumulated ethanol for 15 days (EtOH fed = 10-15, EtOH fed+A.muc = 10, pair fed = 4, pair fed+A.muc = 4)	After liver injury was verified by alanine transaminase (ALT) measurement on day 9, *A. muciniphila* 1.5 × 10^9^ CFU/200 μl orally administered on days 10, 12, and 14	Ethanol-induced intestinal *A. muciniphila* depletion could be restored by oral supplementation	*A. muciniphila* supplementation ameliorated established ALD ↓Liver-to-body-weight ratio, Serum ALT, IL-1β, TNF-α, MPO^+^ cells/HPF, triglyceride, steatosis	Grander et al., 2018
Patients with compensated alcoholic cirrhosis *n* = 12	*Lactobacillus casei Shirota* 3 times per day for 4 weeks	NA	↑Neutrophil phagocytic capacity ↓IL10 ↓TLR4 expression	Stadlbauer et al., 2008
Hospitalized alcohol abstinence patients with alcoholic hepatitis *n* = 117 (probiotics 60 and placebo 57)	1,500 mg *Bacillus* subtilis and *Enterococcus faecium* (formerly known as *Streptococcus faecium*) for 7 days	↓*Escherichia coli*	↑Liver function, systemic inflammation, and endotoxemia	Han et al., 2015
1 year follow-up of male patients with steroid-resistant alcoholic hepatitis *n* = 8	Daily FMT from several donors *via* a nasojejunal tube for 7 days	↓*Proteobacteria* ↑*Actinobacteria* ↓Potentially pathogenic species	Induces of liver disease severity improved significantly within the first week after FMT ↑Survival and liver function	Philips et al., 2017
3 months follow-up of male patients with alcoholic hepatitis treated with: FMT *n* = 16 Pentoxifylline *n* = 10 Corticosteroids *n* = 8 Nutritional therapy *n* = 17	FMT daily *via* a nasojejunal tube for 7 days compared with corticosteroids, nutritional therapy and pentoxifylline	Changes in microbial function and composition were found after FMT ↑*Clostridia* ↑*Bacteroidia* ↓*Gammaproteobacteria* ↓*Bacilli* ↑*Erysipelotrichi*	Survival at 3 months of follow-up was highest in the FMT group	Philips et al., 2018
5 years follow-up of cirrhotic patients received the same treatment with/without rifaximin (*n* = 23/*n* = 46)	4 weeks treatment of rifaximin 400 mg tid	NA	↓Risk of developing variceal bleeding, hepatic encephalopathy, spontaneous bacterial peritonitis and hepatorenal syndrome ↑Five-year cumulative probability of survival	Vlachogiannakos et al., 2013
13 patients with alcohol-related cirrhosis and ascites before and after treated by rifaximin	Oral rifaximin 1200 mg/day for 4 weeks	NA	↓Endotoxin, IL-6, and TNF-α	Kalambokis et al., 2012
Alcohol use disorder (AUD)-related cirrhosis patients (AUDIT-10 > 8) *n* = 20 (FMT *n* = 10 and Placebo *n* = 10)	FMT enema from a donor enriched in Lachnospiraceae and Ruminococcaceae	↑Lachnospiraceae ↑Ruminococcaceae	↓Alcohol craving and consumption ↓Serious adverse events	Bajaj et al., 2021

## The Role of the Microbiome–Gut–Brain Axis in Alcohol-Induced Anxiety and Depression

Psychiatric disorders, such as depression, bipolar disorders, and anxiety disorders, preceded the onset of alcohol abuse, and chronic alcohol exposure induced anxiety and depression symptoms, further aggregating AUD. Alcohol-induced anxiety and depression can bidirectionally interact with gut microbiome (Carbia et al., 2021). As summarized in Table 3, a series of studies from the same research group showed that only part of the alcohol-dependent patients showed dysbiosis in gut microbiome, while the dysbiosis-induced gut leakiness was highly correlated with alcohol craving, alcohol-induced depression, anxiety, and possibility of relapse. Low-grade inflammation in alcohol-dependent patients was also found to be correlated to depression and alcohol craving. In addition, the levels of *Bifidobacterium* spp. and *Lactobacillus* spp. increased significantly in alcohol-dependent subjects with high intestinal permeability during withdrawal period and returned to the level of control group. Besides, the intestinal permeability of alcohol-dependent patients completely recovered after a 3-week withdrawal. Blood lipopolysaccharides produced from gut microbiome increased in alcohol-dependent patients, which can stimulate specific inflammatory pathways correlated to alcohol craving, and gut leakiness favored the translocation of lipopolysaccharides from gut into the circulation system (Leclercq et al., 2012, 2014a,b, 2020). Direct evidence was from preclinical studies. One study established a chronic alcoholism withdrawal mouse model and transplanted gut microbiome of alcoholism and withdrawal mice into normal mice. The results came out that the normal mice developed anxiety behaviors caused by alcohol withdrawal (Xiao et al., 2018). Another study established an animal model of chronic alcohol exposure and innovatively designed three FMT schemes to explore the potential effects of three healthy donors’ fecal microbiome on alcohol-induced neuropsychological behaviors (Xu et al., 2018). Alcohol-induced anxiety and depression in mice can be relieved by transplantation of gut microbiome from healthy human donors, and conversely, healthy mice developed behavioral signs of alcohol withdrawal-induced anxiety by FMT from alcohol-exposed mice (Xiao et al., 2018; Xu et al., 2018). In 2019, Xu’s team used the same mouse model and control group to investigate the gut microbiome and anxiety/depression-like behavior testing, and found increases in *Actinobacteria* and *Cyanobacteria* at the phylum level and *Adlercreutzia* spp., *Allobaculum* spp., and *Turicibacter* spp. at the genus level, but *Helicobacter* spp. decreased. Meanwhile, the expression of BDNF/Gabra1 decreased in the prefrontal cortex (Xu et al., 2019). This team also found that the abundance of *Firmicutes* increased while *Bacteroidetes* decreased in alcoholism, and mice receiving FMT from these patients showed anxiety/depression-like behaviors (Zhao et al., 2020). Another research found that after 2 weeks of antibiotic pre-treatment, the level of *Firmicutes* decreased importantly and the levels of *Bacteroidetes* and *Verrucomicrobia* increased, accompanied by increased alcohol intake and relief of anxiety symptoms during alcohol withdrawal (Reyes et al., 2020). An interesting study collected fecal samples from human donors of alcohol use disorders who showed higher depression scores and alcohol craving with lower *F. prausnitzii* and *A. muciniphila.* After 45 days, AUD FMT mice showed decreased social behavior, more depression-like behavior, and higher cortisol level indicating higher stress, surprisingly with significantly higher *A. muciniphila*, indicating that there might be a comprehensive interaction among gut microbiome, alcohol use disorders, depression, and anxiety behaviors, or an interaction among different species in gut microbiomes (Leclercq et al., 2020). These studies presented a complex interaction among gut microbiome, alcohol exposure, and alcohol-induced anxiety/depression. It might be a challenge as well as a chance, for it was difficult to distinguish the impact of alcohol craving and alcohol-induced anxiety/depression on gut microbiome. Meanwhile, the treatment that relieved alcohol-induced anxiety/depression could also reduce alcohol craving.

**TABLE 3:** Interventions altering the gut microbiome in alcohol-related depression and anxiety.

Recipients	Manipulation	Change in microbiome	Change in behaviors	Change in other system	Citations
Alcohol-dependent patients *n* = 60 (high IP *n* = 26 and low IP *n* = 34) Control group *n* = 15	3 weeks of detoxification and rehabilitation program	Only high IP group showed dysbiosis ↓Overall bacterial load: ↓Ruminococcaceae family ↑Lachnospiraceae family ↑*Blautia* Not in low IP patients	High IP group: remained anxiety, depression, and alcohol craving even after 3 weeks abstinence Low IP group recovered	NA	Leclercq et al., 2014b
3-week-old mice pretreated by antibiotics and polyethylene glycol were performed FMT and behavioral testings (FMT-Alc, FMT-Con *n* = 12/group)	FMT from alcohol-dependent patients every other day in a total of 7 days and 3 times	↓*F. prausnitzii* ↑*A. muciniphila* ↓α-diversity ↓Total bacterial load ↑Relative abundance of *Verrucomicrobia*	↓Social behavior ↑Depressive-like behavior ↑Stress level No difference in anxiety-like behavior and locomotor activity	Attenuated immune system (altered immune responses of Th2 and Th17 cells) Loss of intestinal homeostasis (↓Reg3g and Lcn2) Disturbances of brain function including myelination, neurotransmission, and inflammation	Leclercq et al., 2020
Healthy mice (Water group *n* = 12; Alcohol group *n* = 12; Saline group *n* = NA; FMT group *n* = 5)	FMT from alcohol-exposed mice for 14 days	↓*Bacteroides* ↑*Erysipelotrichia* ↓*Lactobacillaceae* ↓*Bacilli* ↓*Parabacteroides* ↓*Alloprevotella* ↑*Blautia*	Developed depression-like behaviors	Alcoholism-relating genes: ↓BDNF and CRHR1 ↑Oprm1	Xiao et al., 2018
Mice with chronic alcohol exposure (Control group, Alcohol group, FMT group *n* = 6-7 per group)	FMT from 3 young male physically and mentally healthy volunteers for 2, 4, or 5 weeks	NA	5 weeks rather than 2 weeks FMT alleviated alcohol-induced anxiety and depression	NA	Xu et al., 2018
Alcohol-treated group *n* = 18 Control group *n* = 10	Increased concentration of alcohol in their drinking water from 2, 4, to 6% every 3 days, and finally reached 8% in a total of 21 days	↑*Actinobacteria* and *Cyanobacteria* ↑*Adlercreutzia* spp., *Allobaculum* spp., and *Turicibacter* spp. ↓*Helicobacter* spp.	Alcohol-induced anxiety/depression-like behaviors	Decreased GABA1 and BDNF levels correlated with behaviors change	Xu et al., 2019
Mice pretreated by antibiotics (FMT-Alc *n* = 13-14, FMT-Con *n* = 11-12)	FMT from patients with alcoholism every other day in a total of 13 days and 7 times	↓*Bacteroidetes* ↑*Firmicutes*	↑Alcohol preference ↑Anxiety/depression-like behaviors ↓Social interaction behaviors	↓BDNF, α1GABAAR in mPFC ↓mGluR1, PKCε in Nac	Zhao et al., 2020
Mice ethanol consumption behavior were tested in a binge-like “Drinking in the Dark” model for 6 weeks (Water groups-H2O, H2O-20E, ABX groups-ABX, ABX-20E. *n* = 8/group)	2 weeks pretreatment of ABX in drinking water	↑*Bacteroidetes* ↓*Firmicutes* ↑*Verrucomicrobia*	↓Anxiety-like behavior during ethanol withdrawal period ↑Ethanol consumption *Firmicutes* negatively while *Bacteroidetes* and *Verrucomicrobia* positively correlated to ethanol intake levels	NA	Reyes et al., 2020

In sum, the change of gut microbiome is closely related to alcohol-related disorders. However, currently the relationship between alcohol-related disorders and changes in gut microbiome is not consistent. They might be confounded by lots of factors, including different species, alcohol concentration and types of ferment, mode and duration of alcohol administration, stages of AUD, and the duration of withdrawal. Additionally, alcohol caused complicated symptoms accompanied by liver damage, depression, and anxiety, which, in turn, affect gut microbiome, making this field more mysterious. The interactions among gut microbiome–AUD–ALD–alcohol-related/induced depression and anxiety are complicated, and lots of confounding factors could not be easily controlled in research; the alteration in gut microbiome was diverse and inconsistent with each other. However, gut microbiome was irreversible, and after cessation of alcohol exposure or intervention such as probiotics, antibiotics, and FMT, alcohol-induced gut microbiome imbalance can be partially reconstructed and restored. Meanwhile, it can also improve alcohol-induced endotoxemia, liver function impairment, intestinal permeability, alcohol craving, and psychiatric symptoms such as anxiety and depression. Furthermore, some bacteria were considered to directly contribute to alcohol production and metabolism. Due to the persistence and adjustability of gut microbiome, therapy focused on it may be a promising target to treat alcohol-related disorders.

## The Role of the Microbiome–Gut–Brain Axis in Opioid Use Disorders

Studies on the relationship between opioid use disorders and gut microbiome are accumulating, although much fewer than those of alcohol (shown in Table 4). Preclinically, five studies employing mice implanted morphine pellets (Meng et al., 2013, 2015; Banerjee et al., 2016; Wang F. et al., 2018, 2020; Wang G. H. et al., 2018); one study of mice (Zhang L. et al., 2019), one study of rats (Zhang et al., 2020), and one study of rhesus macaques (Sindberg et al., 2019) with injected morphine showed the significantly altered composition of microbiome, but they showed inconsistent results. Intermittent morphine injection and sustained morphine-implanted pellet showed a different impact on gut microbiome in the same way (Lee K. et al., 2018). The discrepancy could be attributed to the limited sample size per group and confounded by the type of administration, dose and duration of morphine exposure, etc. Clinical studies also showed diverse results. A study recruited 48 healthy controls and 45 patients with substance use disorders. All of these patients with daily alcohol and tobacco were poly-drug abusers, including heroin or methamphetamine (MA) and/or ephedrine. The study found that the diversity indexes of patients were higher than controls, with increases in *Prevotella, Paracoccus*, and *Thauera* and a decrease in Bacteroides (Xu et al., 2017). Vincent et al. found that in 90 patients with neither *C. difficile* infection nor colonization, 25 patients who used opioid showed increased diversity of gut microbiome (Vincent et al., 2016). Acharya et al. in 2017 recruited two cohorts with/without opioid use from inpatients and outpatients, respectively, and found a significant dysbiosis in opioid use patients, especially those with hepatic encephalopathy (Acharya et al., 2017). Barengolts et al. conducted a cross-sectional study among African American men with obesity and type 2 diabetes comorbid psychiatric and opioid use disorders. Microbiome analysis showed that *Bifidobacterium* and *Prevotella* could be significantly affected by opioids and metformin interaction (Barengolts et al., 2018). These studies indicated the complex nature of gut microbiome especially in the studies of substance use disorders; more solid and direct evidence are required to figure out the relationship between opioid use disorders and gut microbiome.

**TABLE 4:** Effects of opioid on gut microbiome and other systems.

Species	Opioid administration	Sample size	Effects on microbiome	Effects on microbial metabolites, immune and barrier system	Citations
Human	Active substance users (Heroin Methamphetamine Ephedrine)	Patients with substance use disorders *n* = 45 Control group *n* = 48	↑Species diversity index and the abundance of *Thauera, Paracoccus*, and *Prevotella* No changes specific to heroin, methamphetamine or ephedrine	NA	Xu et al., 2017
	Opioids use	Neither *C. difficile* infection nor colonization *n* = 25 *C. difficile* infection *n* = 3 *C. difficile* colonization *n* = 1	↑Alpha diversity	NA	Vincent et al., 2016
	Opioids use disorders	Cohort I(in-patient): On opioids *n* = 62, Not on opioids *n* = 82. Cohort II(out-patient): On opioids *n* = 72, Not on opioids *n* = 72	↓*Autochthonous taxa* (Ruminococcaceae and Clostridiales *XIV*) and *Bacteroidaceae*	↑Metabolism of aromatic amino acids ↑Degradation of branched-chain amino acids	Acharya et al., 2017
		With opioid use disorder *n* = 45 Without opioid use disorder *n* = 54	↑*Bifidobacterium*	NA	Barengolts et al., 2018
Mouse	Morphine Implanted pellet	25 mg morphine *n* = 5 30 mg naltrexone *n* = 5 Morphine + naltrexone *n* = 5 Placebo *n* = 5	↓Alpha-diversity ↑*Enterococcus faecalis*, *Flavobacterium, Fusobacterium, Sutterella* and *Clostridium*	↓Bile acids ↑Phosphatidylethanolamines and saturated fatty acids	Wang F. et al., 2018
		25 mg subcutaneous morphine sulfate pellet *n* = 7-8 Twice-daily intraperitoneal (i.p.) injections of escalating doses of morphine sulfate (10, 20, 30, 40 mg/kg, *n* = 7–8	Intermittent morphine ↑*Ruminococcus* spp. ↓*Lactobacillus* spp. Sustained morphine ↑Genera *Clostridium* ↑Family *Rikenellaceae*	NA	Lee K. et al., 2018
		Placebo +placebo microbiome *n* = 5 Placebo +morphine microbiome *n* = 5 Morphine + placebo microbiome *n* = 5 Morphine + morphine microbiome *n* = 5	↓*Bacteroidetes, Lactobacillus* and *Clostridium* ↑*Firmicutes (Enterococcaceae, Staphylococcaceae, Bacillaceae, Streptococcaceae, and* Erysipelotrichaceae)	↓Primary and secondary bile acids in the gut not in the liver ↑Level of coprostanol and cholesterol	
		Poly-microbial sepsis mice model induced by cecal ligation and puncture (CLP) treated by 25 mg slow-release morphine pellet Placebo *n* = 5 Morphine *n* = 5 Placebo+CLP *n* = 5 Morphine+CLP *n* = 5	↑*Firmicutes* phylum (specifically the G+ bacterial species, *Staphylococcus sciuri, Staphylococcus cohnii, Staphylococcus aureus, Enterococcus durans, Enterococcus casseliflavus, Enterococcus faecium*, and *Enterococcus faecalis)* ↑Translocation of Gram-positive gut bacteria	↑CLP mice mortality, bacterial dissemination, IL-17A, IL-6.	Meng et al., 2015
		WT and m-opioid receptors (MOR) knockout (MORKO) mice administrated with 75 mg morphine pellets for 24 h *n* = 9–10	Chronic morphine compromises barrier function of gut epithelium Bacterial translocation to mesenteric lymph node (MLN) and liver	Disrupts tight junction organization between small intestinal epithelial cells; Disrupts tight junction organization between small intestinal epithelial cells.	Meng et al., 2013
	15 mg/kg morphine injection b.i.d. for 8 days	Saline group *n* = 6 Morphine group *n* = 7	↓*Actinobacteria* ↓*Firmicutes* ↓Bifidobacteriaceae ↓*Lactobacillaceae*	Disrupted gut epithelial barrier and promoted systemic Bacterial translocation; ↑TLR2 and TLR4 expression; ↑Sustained chronic systemic inflammation.	Zhang L. et al., 2019
Rat	10 mg/kg morphine i.p.	Morphine group *n* = 28 Saline group *n* = 7	No significant differences in alpha diversity Morphine group ↓*Alloprevotella, Desulfovibrio*, and *Rikenella* ↑*Allobaculum* and *Parasutterella* Saline group ↓*Corynebacterium*, *Clostridium_XlVa, Corynebacterium*, and *Parasutterella* ↑*Desulfovibrio*	NA	Zhang et al., 2020
Indian-origin rhesus macaques	Intramuscular injection	Morphine group *n* = 4 Simian immunodeficiency virus (SIV) group *n* = 4 Morphine+SIV group *n* = 6	No significant differences in microbial diversity ↑*Methanobacteriaceae* ↓*Streptococcaceae streptococcus* and *Pasteurellaceae Aggregatibacter*	↓Primary bile acids ↑Secondary bile acids and sphingolipid metabolites	Sindberg et al., 2019

## The Role of the Microbiome–Gut–Brain Axis in Tolerance of Morphine Analgesia

Several animal studies focused on the role of microbiome in an individual’s response to morphine analgesia. Opioid analgesics represented by morphine have been widely used as medication all over the world. However, the long-term medical use of opioid as pain management is limited by its analgesic tolerance. It would be promising if microbiome could be a modulator for an individual’s response to morphine analgesia. Firstly, the study of Kang et al. showed that antibiotics can increase the tolerance to morphine, thus inhibiting the analgesic effect of morphine, comparing the control group with the antibiotic group and the antibiotic–morphine chronic exposure group (Kang et al., 2017). Later, Lee et al. transplanted fecal microbiome from the opioid-dependent group to antibiotics-pretreated mice, significantly increasing the response to morphine analgesia of these mice and restoring their cocaine location preference behavior (Lee K. et al., 2018). In 2018 and 2019, two studies from the same group also reached a consistent conclusion. The germ-free mice or antibiotic pretreatment presented prolonged efficacy of morphine in the treatment of mice and reduced the tolerance of morphine analgesic effect (Wang F. et al., 2018; Zhang L. et al., 2019). The evidence above supported the idea that gut microbiome played an important role in the tolerance to morphine analgesia, and manipulations of gut microbiome by antibiotic treatment may be a potential treatment to improve the response to morphine analgesia. Furthermore, Wang et al. further found significantly increased *Enterococcus faecalis* induced by morphine, and *E. faecalis* colonized mice showed significant increase in morphine tolerance (Wang F. et al., 2018). A further study by Zhang et al. reported that FMT from normal mice did not significantly prevent the occurrence of tolerance to morphine analgesia after chronic morphine exposure, but after 21 days of intragastric pretreatment with probiotic VSL # 3, composed of eight different strains of bacteria mainly belonging to *Bifidobacterium* and *Lactobacillus*, the tolerance significantly decreased. This study further explored the underlying mechanism and found that morphine tolerance was mediated by probiotics through activating TLR2/4 and pro-inflammatory cytokine cascades (Zhang L. et al., 2019). In sum, all evidence supported the idea that gut microbiome, especially *Bifidobacteria* and *Lactobacillus*, played an important role in tolerance to morphine analgesia. In addition to germ-free or antibiotic pretreatment, *Bifidobacteria* and *Lactobacillus* transplantation might be a safer, more effective, and more economical treatment to improve the individual’s response to morphine analgesics. All of these studies are summarized in Table 5.

**TABLE 5:** Interventions altering the gut microbiome in opioid-related disease.

Subjects and manipulation	Changes in microbiome and metabolic and immune system	Changes in addiction-related behaviors	Changes in nociceptive tolerance	Citations
Morphine-pelleted (MP) or placebo-pelleted (PP) mice + oral gavage Antibiotic cocktail (ABX) or different combinations of antibiotics twice a day for 10 days. *n* = 10	↓Overall gut microbiome ↓chronic morphine induced increases in gut permeability and colonic mucosal destruction ↓Colonic IL-1β expression	ABX did not alert on morphine-induced dependence by naloxone-precipitated withdrawal.	↓Morphine analgesic tolerance (oral ABX or vancomycin alone)	Kang et al., 2017
Mice oral antibiotics or antibiotics treatment with morphine (intermittent systemic injections or sustained sub-cu pellet) *n* = 4–6	↓Overall gut microbiome ↑Microglia cell body size	↓Reward response (Cocaine-CPP)	↑Hyperalgesia in antibiotic mice when treated with morphine	Lee K. et al., 2018
Antibiotic-treated mice FMT from naive mice donors or morphine-dependent donors *n* = 6-8	Fecal microbiome diversity following microbiome recolonization	FMT from naive mice donors restores normal reward behavior and microglia morphology in antibiotic-treated mice	FMT from naive mice donors restores sensitive to pain in antibiotic-treated mice	Lee K. et al., 2018
Mice were gavage *E. faecalis* (EF) 2 × 10^10^CFU/ml or PBS for 8 days + Morphine; *n* ≥ 10	NA	NA	↑Morphine analgesic tolerance and hyperalgesia; ↑Hyperalgesia	Wang F. et al., 2018
GF mice or control group+ Morphine. *n* = 4–5 Mice treated by non-absorbable broad-spectrum pan-antibiotics cocktail (ABX) or water for 7 days +Morphine; *n* = 12-20	↓Overall gut microbiome	NA	↓Morphine analgesic tolerance (GF or pan-antibiotic-treated)	Zhang L. et al., 2019
Mice orally treated by probiotic (VSL#3: *Bifidobacteria* and *Lactobacillaeae*) or water for 21 days+Morphine; *n* = 10–20	Substantially restored the bacterial communities that were significantly reduced in relative abundance in morphine-tolerant animals compared with saline control sample	NA	↓Morphine analgesic tolerance Suggested that probiotic pretreatment can prolong the efficacy of morphine as an analgesic agent	Zhang L. et al., 2019

Compared with alcohol-related disorders, studies on the correlation between morphine-related disorders and gut microbiome were much fewer. Similarly, the changes of gut microbiome in morphine-related disorders were also affected by many factors such as different animal models, different routes of administration, duration of exposure, and different stages of the disorders. Most of the studies focused on the role and mechanism of gut microbiome in morphine analgesia. Probiotics mainly containing *Bifidobacteria* and *Lactobacillus* might become a promising therapeutic drug to prolong the analgesic effect of morphine.

## The Role of the Microbiome–Gut–Brain Axis in Psychostimulant Use Disorders

The abuse of psychostimulants, including cocaine and amphetamines, especially novel synthetic drugs, has posed serious impact all over the world. However, studies on the microbiome–gut–brain axis and addiction to psychostimulants were much fewer and mainly based on animal models.

As shown in Table 6, Ning et al. focused on the relationship between MA and gut microbiome, in which a dysregulation of gut microbiome was detected after a 14-day induction of conditioned place preference (CPP) using MA, including a significant decrease of the relative abundance of *Phascolarctobacterium* and a decrease of propionate. What is more, unlike other addictive drugs, the diversity of microbial community in the MA group was slightly higher than that in the control group (Ning et al., 2017). This result has been further verified by our team in 2020, and our study further found the association between gut microbiome and an individual’s response to MA reward property (Yang et al., 2021). Similarly, Kiraly et al. (2016) also found that the sensitivity of mice to cocaine reward and their behavioral response to chronic cocaine use were also enhanced after antibiotic treatment before CPP training. A recent research showed that compared with amphetamine stimulants such as MA and 4-methylmethamphetamine, the effect on gut microbiome in mice treated with selected synthetic psychoactive cathinones (including methoxyephedrine and methylcathinone) was significantly different (Angoa-Perez et al., 2020), which indicates that different substances, even different kinds of psychostimulants, have different effects on gut microbiome, and this may be caused by different pharmacological mechanisms of drugs themselves; nevertheless, many other confounding factors may exist. So far, there was only one clinical study by Volpe et al. that examined the gut microbiome of patients with cocaine addiction and healthy controls, and the results showed that the Bacteroides in patients significantly increased (Volpe et al., 2014). An elegant study showed that a surgery ligating the common bile duct and anastomosis of the gallbladder to the ileum (GB-IL) or voluntary oral administration of semi-synthetic bile acid analog OCA blocked the increased DA levels in the NAc and reduced cocaine locomotor sensitization as well as cocaine CPP, and further demonstrated the mechanism of bile acid signaling in regulating the behavioral response to cocaine *via* Takeda G protein-coupled bile acid receptor (TGR5), utilizing a TGR5 knockout mouse model and viral restoration of NAc, which is sufficient to restore cocaine reward. This study opens up novel ways of gut-to-brain communication (Reddy et al., 2018). As summarized in Table 7, antibiotic-treated mice showed increased cocaine CPP response while antibiotic-pretreated rats showed elevated MA-induced CPP (Yang et al., 2021). In sum, although there is a lack of clinical studies about the role of gut microbiome in psychostimulant use disorders, preclinical studies showed discrepancy in gut microbiome induced by the exposure of psychostimulants, compared to other substances or among different kinds of psychostimulants. Manipulations of gut microbiome by antibiotic treatment consistently showed the increased sensitivity to drug reward and behavioral response to psychostimulant use; additionally, bile acid signaling regulated the behavioral response to cocaine *via* TGR5. These data indicated the potential role of gut microbiome in individual susceptibility of psychostimulants addiction. However, compared to alcohol and opioid, the roles of gut microbiome in psychostimulant use disorders still need to be clarified.

**TABLE 6:** Effects of psychostimulants on gut microbiome and other systems.

Species	Drugs	Sample size	Effect on microbiome and microbial metabolite	Effect on other systems	Citations
Human	Cocaine users	HIV+ Cocaine users *n* = 7 HIV+ Cocaine nonusers *n* = 8 HIV- Cocaine users *n* = 7 HIV- Cocaine nonusers *n* = 10	↑*Bacteroidetes* ↓*Firmicutes*	No significant effect IL-1G, IL-6, 16S rRNA gene and LPS HIV-infected cocaine users had higher interferon-γ levels than all other groups	Volpe et al., 2014
Mice	Mephedrone (40 mg/kg), methcathinone (80 mg/kg), methamphetamine (5 mg/kg) 4-methyl-methamphetamine (40 mg/kg) i.p. 4 injections at 2-h intervals	*n* = 5-7 mice per group	Diverse alteration in α-diversity, β-diversity, relative abundance of OTUs and bacterial phyla level among different drugs as well as at different days after drug injections. BLAST analysis identified individual bacterial species linked to specific drugs at different days: *Fusimonas intestine, Mucispirillum schaedleri* linked to mephedrone at day 1. *Paramuribaculum intestinale* linked to methcathinone at day 1 and mephedrone at day 7. *Duncaniella muris* linked NA to methcathinone at day 1 and 4-methyl-methamphetamine at day 7.	NA	Angoa-Perez et al., 2020
Rat	Methamphetamine Intraperitoneal injection	Methamphetamine group *n* = 8 Control group *n* = 8	↓*Phascolarctobacterium* ↓Propionate ↑Ruminococcaceae, *Bacillaceae, Proteobacteria and Fusobacteria* ↑Fecal microbial diversity No differences in the relative abundance of n-butyric acid	NA	Ning et al., 2017
	Methamphetamine Intraperitoneal injection	Methamphetamine group *n* = 17 Saline group *n* = 5	↑*Akkermansia, Butyricimonas* ↓*Acetivibrio* In higher MA-induced CPP responder rats	NA	Yang et al., 2021

**TABLE 7:** Interventions altering gut microbiome in psychostimulant-related disorders.

Species	Drugs	Microbiome or metabolite manipulation	Behavioral effects	Citations
Mice	Cocaine intraperitoneal injection	Antibiotic+cocaine *n* = 5 Saline+cocaine *n* = 5 SCFA+cocaine *n* = 5–12 SCFA/ABX+cocaine *n* = 5–12	↑Locomotor sensitization and conditioned place preference at lower dose (5 mg/kg) SCFAs *via* drinking water reverses the antibiotic-induced behavioral phenotype.	Kiraly et al., 2016
		Surgical intervention to increase bile acid by ligation of the common bile duct and anastomosis of the gallbladder to the ileum (GB-IL) *n* = 13–14 Or by voluntary oral administration of semi-synthetic bile acid analog OCA *n* = 7–8	↓Locomotor sensitization and conditioned place preference ↓Cocaine-induced DA levels in the NAc ↓Conditioned place preference	Reddy et al., 2018
Rat	Methamphetamine (MA) intraperitoneal injection	Antibiotic+MA *n* = 8 Saline+MA *n* = 8	↑Conditioned place preference	Yang et al., 2021

## The Role of the Microbiome–Gut–Brain Axis in Other Substance Use Disorders

Other addictive substances, such as nicotine (Chi et al., 2017), marijuana (Panee et al., 2018), and ketamine (Yang et al., 2017), also have an impact on the gut microbiome. A population-based study found significant differences in gut microbiome between current smokers and non-smokers (Lee S. H. et al., 2018; Nolan-Kenney et al., 2020). Research has proved that nicotine, as the main active content in tobacco, has effects on gut microbiome community composition, functional bacterial genes, and fecal metabolites (Chi et al., 2017). Similarly, preliminary results demonstrated a close correlation between the use of cannabis and the change of gut microbiome, such as a significantly increased *Prevotella*:*Bacteroides* ratio, which may further lead to cognitive impairment (Panee et al., 2018). Chronic Δ^9^-tetrahydrocannabinol (THC)-administrated mice showed altered gut microbiome composition (Cluny et al., 2015). Furthermore, studies showed that cannabinoid cannabidiol (CBD) or THC showed preclinical promising in alcohol use disorders and diet-induced obesity collectively through the microbiome–gut–brain axis (Cluny et al., 2015; Cani et al., 2016; Karoly et al., 2020). Ketamine, an inhibitor of the central nervous system, also has specific effects on the levels of *Mollicutes* and *Butyricimonas*, and may exert its antidepressant effect through the microbiome–gut–brain axis (Yang et al., 2017). These lines of evidence suggest that gut microbiome may contribute to substance use disorders through the microbiome–gut–brain axis, providing a new perspective for the study of addiction mechanism and therapeutic target investigation.

## The Role of the Microbiome–Gut–Brain Axis in Individual Susceptibility of Addiction

Through studies about the correlation between addiction and gut microbiome, it turns out that gut microbiome plays a role in individual’s susceptibility of addiction. The susceptibility of addiction appears that, after an initial exposure to the same dose of the same addictive substance, some individuals remain in the stage of occasional use for a long time, while some gradually develop into regular use, and finally compulsive drug-seeking and addiction (Hao et al., 2016). One study found that uncontrollable drug-seeking behavior only occurred in 15% of the mice with alcohol self-administration. According to the behavioral indicators related to alcohol addiction, these rats were divided into alcohol-susceptible groups and alcohol-resistant groups. Comparing the gut microbiome between the two groups, significant differences were found, which was related to the response of rats to alcohol. The relative abundances of Clostridiales, Ruminococcaceae, and Lachnospiraceae were positively correlated with alcohol-use behavior, while the relative abundances of *Desulfovibrio*, *Gemella*, Coriobacteriaceae, and *Hydrogenoanaerobacterium* were negatively correlated with total alcohol consumption (Jadhav et al., 2018). In 2020, a study from Reyes et al. found that after 2 weeks of antibiotic pretreatment, the spontaneous alcohol intake of mice in the drinking in the dark model increased significantly, and the intestinal *Bacteroides* increased while *Firmicutes* decreased (Reyes et al., 2020). These findings suggest that gut microbiome is significantly associated with individual susceptibility to alcohol, and changing gut microbiome can change an individual’s susceptibility to alcohol. Furthermore, Bareli’s study on rats showed that cocaine self-administration altered gut microbiome, while dehydroepiandrosterone treatment, an endogenous neurosteroid and a food supplement, attenuated drug-seeking behavior by shifting the gut microbial diversity towards the control group and raised a certain bacterium, then reduced the drug-seeking behavior of rats (Bareli et al., 2019). Kiraly et al. (2016) found that the sensitivity of mice to cocaine reward and their behavioral response to chronic cocaine use were also enhanced after antibiotic treatment of mice before CPP training. In addition, Lee et al. found that after intermittent morphine administration, antibiotic therapy can reduce opioid analgesic efficacy and damage brain cocaine reward circuit by reproducing neuroinflammation, and reduce the CPP value of cocaine in mice. To further verify the experimental results, they colonized microbiome of untreated normal controls in mice treated with sustained antibiotics, and found that it can help restore the activation state of microglia and the CPP behavior to cocaine in mice (Lee K. et al., 2018). Our team found that only part of the rats exposed to the same dose of MA showed a significant preference for drug testing in CPP training. Furthermore, comparing the changes of gut microbiome in rats grouped with high MA preference and low MA preference, we found that *Verrucomicrobia* and *Butyricimonas* increased significantly in the high-preference group, and the relative abundance was positively correlated with the increased preference, while *Acetivibrio* increased significantly in the low-preference group, and was correlated with the decreased preference. Furthermore, rats pretreated with broad-spectrum antibiotics for 1 week showed a significantly increased MA preference (Yang et al., 2021) (see Table 7). Following a similar strategy, we found that the altered bacterial phyla level also correlated with morphine-induced preference (Zhang et al., 2020). These lines of evidence suggested the important role of gut microbiome in individual susceptibility of addiction or behavior response to alcohol, opioid, cocaine, and MA. Manipulation of gut microbiome might be an efficient modulator for individual sensitivity to drug award, morphine analgesia, and risk of development of addiction. However, this field is still under development, and more direct and strong evidence from both clinical and preclinical studies are required.

## Tridirectional Interaction Among Gut Microbiome, Substance Addiction, and Substance-Induced/Related Mental Disorders

Substance use disorder is a comprehensive disorder with lots of symptoms overlapping with other psychiatric disorders. First of all, various psychotic symptoms induced by substance use usually appear during or immediately after substance use (within 48 h); onset excluding delirium and withdrawal symptoms or after 2 weeks of substance use can be diagnosed as mental disorders caused by substance use in DSM-5, including psychotic disorders, bipolar and related disorders, depressive disorders, anxiety disorders, sleep disorders, sexual dysfunction, delirium, and neurocognitive disorders. Secondly, the comorbidity in substance use disorder and mental disorders is also very common. According to the National Comorbidity Study, 50.9% of Americans who have suffered from lifelong mental disorders have also suffered from substance abuse. Meanwhile, among Americans who have suffered from lifelong drug abuse, the lifetime prevalence of mental disorders is 51.4%. Mood disorders, anxiety disorders, and personality disorders are more common (Hawkins, 2009; Green and Drake, 2011). As mentioned above, alcohol-induced anxiety/depression could be produced by FMT from alcohol-exposed donors and relived by probiotic treatment (Reyes et al., 2020; Zhao et al., 2020). In a study by Reyes et al. (2020), 2 weeks of antibiotic pretreatment relieved anxiety symptoms during alcohol withdrawal, and the level of *Firmicutes* negatively correlated while *Bacteroidetes* and *Verrucomicrobia* positively correlated to alcohol intake. Plenty of other addictive substances, such as MA or ketamine, are frequently reported as various psychotic symptoms (McKetin et al., 2017), while tons of clinical and basic studies have proved the interaction between these mental disorders and gut microbiome (Sharon et al., 2016; Barbosa and Vieira-Coelho, 2020; Chinna Meyyappan et al., 2020; Zagorska et al., 2020). How could we rule out the confounding effects of substance-induced/related mental disorders when we focus on the correlation between gut microbiome and substance addiction? Is there any commonality or difference about the role of gut microbiome in mental disorders and substance-induced/related mental disorders? Could the microbiome manipulation that provides relief for alcohol-induced anxiety/depression be a treatment for anxiety/depression disorders? Further investigations are required to answer these questions.

## Challenge and Prospect

**FIGURE 1:**
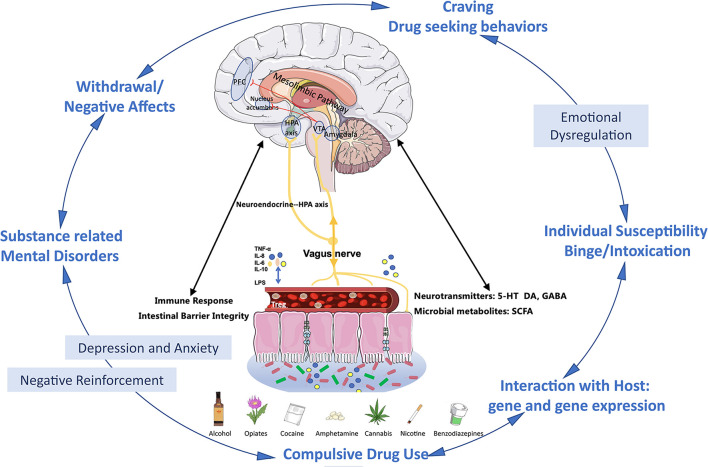
Microbiome–gut–brain in substance use disorders and substance-related mental disorders. First, via the microbiome–gut–brain axis, substance exposure-induced dysbiosis leads to the damage of the gut epithelial barrier. In turn, it further causes the increased permeability (“leaky gut”) of bacterial products such as LPS lipopolysaccharide (LPS), immune cytokines (TNF-α, IL-6, IL-8, IL-10, etc.), hormones, neurotransmitters, and microbial metabolites produced by the gut microbiome. These products can be released to the systemic circulation such as blood stream and then transported to the brain, and these gut derived components also target the HPA axis and vagus nerve. These all together further impact on brain barrier integrity and immunoactivities in brains and alter the reward system (Carbia et al., 2021; Garcia-Cabrerizo et al., 2021). Second, the gut microbiome plays an important role in the cycle of development and maintenance of addiction. At the binge/intoxication stage, substance use is mainly initiated by individual susceptibility of the drug, which might be determined by the interaction between the microbiome and the host genetic background. In this stage, the same emotional problems might also precede the use of the drug. Repeated binge induces the progressively microbiome imbalances and contributes to early emotional dysregulation, and further motivates the craving and drug seeking behaviors. At the withdrawal/negative effect stage, exaggerated emotional disturbances and dysbiosis combine with the negative effects related to withdrawal driven negative reinforcement and finally accelerate the transition to compulsive alcohol use (Wise and Koob, 2014; Carbia et al., 2021; Garcia-Cabrerizo et al., 2021). Additionally, the substance-related mental disorders, especially depression and anxiety, could further exacerbate dysbiosis (Reyes et al., 2020; Carbia et al., 2021). Abbreviations: PFC, prefrontal cortex; VTA, ventral tegmental area; NAc, nucleus accumbens; AMYG, amygdala; NTS, nucleus tractus solitarii; IL, interleukin; TNF, tumor necrosis factor; LPS, lipopolysaccharide; SCFA, short-chain fatty acids; 5-HT, 5-hydroxytryptamine; DA, dopamine; GABA, γ-aminobutyric acid; HPA, hypothalamic–pituitary–adrenal.

Research on gut microbiome and substance-related disorders is promising but still under development. As showed in Figure 1, present evidence reveals that gut microbiome plays an important role in substance use disorders *via* the microbiome–gut–brain axis, and preclinical studies suggest the therapeutic potential of gut microbiome manipulation in substance-related disorders, which has a very promising future. However, more evidence especially from clinical studies is needed and there are lots of challenges to further demonstrate the causal relationship between gut microbiome and substance-related disorders, to investigate the underlying mechanism and explore therapeutic targets.

### Confounding Factors Complicate the Problem

Substance use disorders are comprehensive disorders. There might be lots of confounding factors in the study of gut microbiome and substance use disorders. On the one hand, firstly, gut microbiome itself could be affected by various factors including age, gender, genetic background, diet, infection and diseases, medications, and stressors. Secondly, specifically, there are several substance use-related confounding factors, such as different addictive substances, different dosages and duration of the same substance, at different stages of addiction (acute use, chronic use, withdrawal and relapse, etc.), different combinations and dosages of antibiotics/probiotic treatment, liver function damage, endotoxemia, and other systemic diseases such as HIV or bacterial infection accompanied or induced by the use of substances. Additionally, other physical diseases, psychotic symptoms, or psychiatric disorders such as schizophrenia, mood dysfunction, anxiety neurosis, or personality disorders associated with substance use also interacted with gut microbiome. All of the above factors complicated this problem. On the other hand, there is a very comprehensive mechanism in substance-related disorders, and what we can see is based on a one-sided viewpoint. Firstly, it is likely that there are multiple gut microbiomes rather than a single bacterium that plays an essential role in substance-related disorders. Gut microbiome, host genetic background, gene expression difference, and other factors combined together affect the onset and development of substance-related disorders. Therefore, to further study the role of gut microbiome in substance-related disorders, well-designed experiments, adequate sample size, and proper analysis are needed.

### Possibility of Shared Biomarkers in Gut Microbiome for Different Substance Addiction

It is worth mentioning that different substance-related disorders have common behavioral characteristics and neurobiological mechanisms, including irrepressible and uncontrollable drug craving and repeated compulsive drug-seeking behavior related to drug addiction (Hao et al., 2016). The common neural structural basis of addiction is the VTA–NAc circuit of reward circuit located in the limbic system (Kim et al., 2016; Koob and Volkow, 2016). Long-term abuse of addictive substance can lead to abnormal activation of dopaminergic neurons in reward circuits (Koob and Le Moal, 2001). There will also likely be a common specific pattern about correlated changes of gut microbiome in different addictive substance-related disorders when the variables are strictly controlled. It will not only identify novel common biomarkers of gut microbiome for substance addiction but also become a collective therapeutic target for substance use disorders.

## Summary and Conclusion

Current studies illustrate a complex relationship between gut microbiome and substance-related disorders, involving multiple confounding factors. Also, gut microbiome may interact with the genetic background and gene expression of host cells to influence the occurrence and development of substance-related disorders. However, plenty of preclinical animal studies have found that different gut microbiome of different individuals or the change of gut microbiome will in turn affect the host, including the response to addictive substances and the onset and development of substance-related/induced mental diseases. These lines of evidence suggest that gut microbiome plays an important role in substance-related disorders and has a potential to be a new therapeutic target. However, it is still lacks strong and direct evidence especially from clinical trials to make a final conclusion. In a word, the research on the relationship between substance use disorder and gut microbiome is still in its infancy with a bright future ahead, but there is a long way to go.

## Author Contributions

XZ designed the overall idea. XZ, XF, TC, and JC performed the review of the literature and wrote the manuscript. BL and YZ participated in reviewing the manuscript. All authors contributed to the article and approved the submitted version.

## Conflict of Interest

The authors declare that the research was conducted in the absence of any commercial or financial relationships that could be construed as a potential conflict of interest.

## Publisher’s Note

All claims expressed in this article are solely those of the authors and do not necessarily represent those of their affiliated organizations, or those of the publisher, the editors and the reviewers. Any product that may be evaluated in this article, or claim that may be made by its manufacturer, is not guaranteed or endorsed by the publisher.
